# Blended Delivery of a Primary Care Parenting Program for Child Development: A Randomized Clinical Trial

**DOI:** 10.1001/jamanetworkopen.2025.56024

**Published:** 2026-02-03

**Authors:** Susan M. Chang, Joanne A. Smith, Amika S. Wright, Julia Rowe-Porter, Kemisha Shaw-Kelly, Florencia Lopez-Boo, Susan P. Walker

**Affiliations:** 1 Caribbean Institute for Health Research, The University of the West Indies, Kingston, Jamaica; 2 Family Health Unit, Ministry of Health & Wellness, Kingston, Jamaica; 3 Inter-American Development Bank, Washington, DC; 4 Global TIES for Children, New York University, New York; 5 Steinhardt School of Culture, Education, and Human Development, New York University, New York

## Abstract

**Importance:**

There is substantial evidence that programs that build parents’ abilities to support early learning benefit children’s development. Evidence is needed on how to integrate and scale these programs within government services.

**Objective:**

To determine whether a parenting program implemented by government primary health care services and combining in-person and remote delivery benefits children’s development and parenting attitudes and behaviors.

**Design, Setting, and Participants:**

In this single-blind randomized clinical trial, enrollment began in July 2022 in primary health care centers in Jamaica. The intervention was phased in from September 2022 to August 2023 and lasted 8 months. Data collection ended in April 2024. Families with children aged 3 to 28 months from communities served by the centers were identified by health staff. Following eligibility checks and informed consent, families were randomly assigned to intervention or waiting list control.

**Intervention:**

The intervention aimed to strengthen parents’ skills in helping their child learn through play and responsive interactions. Community health workers used a curriculum with age-appropriate play and language activities and made fortnightly contacts with families, alternating between home visits and telephone calls.

**Main Outcomes and Measures:**

Child development was measured with the Griffiths Mental Development Scales and parenting behaviors (involvement, responsivity, acceptance, and learning materials) with the Home Observation for Measurement of the Environment (HOME). Effect size (ES) was calculated by dividing the regression coefficient by the pooled-sample SD.

**Results:**

A total of 627 children were enrolled (311 intervention and 316 control; 322 [51.4%] male). At follow-up, 491 children (78.3%) were assessed, 237 (76.2%) of those in the intervention group and 254 (80.4%) in the control group. Children’s mean (SD) age at follow-up was 27.0 (5.8) months in the control and 27.0 (6.1) months in the intervention group. A total of 393 mothers (62.7%) had completed secondary school. There were no significant differences in follow-up rate or characteristics between groups. Intention-to-treat multivariate regression analyses with inverse probability weights showed benefits for children’s overall developmental quotient (ES, 0.17 SD; 95% CI, 0.01-0.33 SD), fine motor ability score (ES, 0.19 SD; 95% CI, 0.03-0.36 SD), and parent behaviors (HOME score ES, 0.25 SD; 95% CI, 0.08-0.41 SD).

**Conclusions and Relevance:**

In this randomized clinical trial of a parenting program delivered through primary health care, the intervention benefited child development and parenting. Findings suggest combining in-person and remote methods may be a useful strategy for scaling.

**Trial Registration:**

ISRCTN Registry Identifier: ISRCTN11059214

## Key Points

**Question:**

Does a parenting program combining in-person and remote delivery and implemented by a government primary health system benefit early childhood development and parent behavior?

**Findings:**

In this randomized clinical trial of 627 parent-child pairs in Jamaica, children in the intervention group had significantly higher developmental levels and their parents provided more stimulating home environments compared with controls.

**Meaning:**

The findings suggest combining in-person and remote delivery methods is a promising strategy for scaling early childhood development programs.

## Teaser

This randomized clinical trial explores whether blended in-person and remote delivery of an early childhood parenting program benefits child development and parenting practices among caregiver-child dyads in Jamaica.

## Introduction

Responsive caregiving and opportunities to learn are critical components of care, as indicated in the World Health Organization’s Nurturing Care Framework.^1^ Guidelines for improving early childhood development (ECD) call on governments to provide support services for families who are the main providers of care for young children.^2^ There is consistent evidence that building parent skills to promote development benefits parenting behaviors and children’s development.^3,4,5,6^ There is also evidence that offering ECD programs with other services for families, such as health and social protection, may be suitable strategies for scaling.^7,8,9,10,11,12^

The Reach Up early childhood parenting program was developed to increase capacity for implementation of ECD parenting programs in low- and middle-income countries and provides curricula, comprehensive training materials, and manuals on toy making, adaptation, and planning.^13^ The program has been implemented in several countries, and a meta-analysis of evaluations across 8 countries showed significant benefits for children’s cognitive, language, and fine motor abilities and the quality of the home environment.^14^

The COVID-19 pandemic accelerated development of remote delivery methods for ECD programs. Experiences in Latin America and the Caribbean indicated that remote delivery facilitated continued delivery of services^15^ and was accepted by the ECD workforce and families.^16^ However, more evidence is needed on the effectiveness of remote delivery methods and whether these methods may facilitate scaling. Evidence before the pandemic showed promise,^17,18^ with mixed findings during the pandemic. Remote delivery benefited parenting practices in Jamaica,^19^ but there were no significant associations with ECD or parenting practice when text and audio messaging were sent to families in Uruguay.^20^ A telephone-based intervention for Syrian and Jordanian families reduced maternal depressive symptoms but had no significant effects on other parenting outcomes (including parent-child learning activities) or child development.^21^ Interviews with health staff and families in Jamaica suggested remote methods were feasible and acceptable, although staff felt that some in-person contact was necessary.^19^

Combining remote and in-person delivery could facilitate scaling. The objective of this study was to determine whether the Reach Up program, delivered by government primary health care services using a blended approach, benefited children’s development (primary outcome) and parenting behaviors. A second objective was to determine whether impact varied by child’s age and sex, mother’s educational level, or maternal depressive symptoms.

## Methods

The Reach Up program is implemented in Jamaica by the Ministry of Health & Wellness (MOHW). In 2019, each parish health team (13 parishes) nominated a health district to participate. Districts generally comprise 3 to 7 primary care centers. Six parishes began fortnightly home visits delivered by community health workers (CHWs) before visits were suspended in March 2020 due to the COVID-19 pandemic. Remote delivery was implemented in these 6 parishes in September 2020 and evaluated from July to October 2021.^19,22^ For this single-blind randomized clinical trial, we recruited a new evaluation sample from the health districts originally nominated. The study was approved by the University of the West Indies research ethics committee (protocol in Supplement 1). Written informed consent was obtained from each child’s primary caregiver before data collection. Participants were advised that participation was voluntary, and they could withdraw from the study at any time. Data were kept in a locked file cabinet or password-protected computer, and access was limited to research staff. The results reported herein adhere to the Consolidated Standards of Reporting Trials (CONSORT) reporting guideline.

### Sample

Families were eligible if they had a child aged 3 to 28 months and were beneficiaries of the Jamaica conditional cash transfer program or known by the health staff to live in low-income circumstances. Exclusion criteria were maternal age less than 18 years, due to requirement for adult consent; a child who attended daycare; no consistent caregiver at home; or a child who had a disability likely to affect development.

Families were identified by CHWs and nurses. The research team conducted eligibility checks and collected informed consent from the primary caregiver. All children received usual care at the health centers (eg, well-child checks, immunization).

### Sample Size

To enable the CHWs to conduct the intervention together with their usual duties, the number of families enrolled per CHW was limited to 8, with 4 families randomly assigned to intervention and 4 to the waiting list control group. With approximately 8 CHWs per health district, we estimated 32 children in each group per district, giving 416 per group across all 13 districts.

Recruitment was not possible in 1 health district, reducing the estimated total to 384 per group. Effect size (ES) was calculated by dividing the regression coefficient by the pooled-sample SD. From prior work,^23^ we estimated an ES for child developmental quotient (DQ) of 0.3 SD (ie, 3.6 points [SD, 12 points]); the total DQ range for ages 0 to 2 years is 60 to 140 points, with lower scores suggesting delayed development.^24^ A sample size per group of 178 was required for 80% power at a significance level of *P* < .05. The estimated sample of 384 allowed for lower recruitment levels in some districts and loss to follow-up.

### Description of Intervention

The Reach Up program works through strengthening parents’ skills and enjoyment in helping their child learn through play and responsive interactions. The intervention curriculum provides age-appropriate activities. Children begin at their age level and usually move on to the next activities with each contact. The curriculum suggests ways to adjust activities depending on the child’s ability. CHWs made fortnightly contacts by home visits or telephone calls, conducted alternately. Details of the intervention are provided in the Box.

Box. **Description of Intervention**

**Type of Community**

Three urban and 9 rural health districts

**Implementing Agency**

Ministry of Health & Wellness, Jamaica

**Supervisors**

Public health nurses and midwives

**Delivery Agents**

CHWs

**Intervention Content Received by Caregivers**

Parent handbook with brief descriptions and pictures of language and play activities
Home visit or telephone call alternately every 2 wk
Play materials, provided once per month at home visit
Weekly text messages

**Outline of Contacts**

Contacts began with a brief interaction about how the caregiver and child had progressed with previous activities.
Home visits: The CHW introduced new activities by first observing what the child did, then demonstrating and describing the activity to the caregiver and child; encouraging the child to try it and offering help if needed; and then encouraging the caregiver and child to do the activity together. CHWs demonstrated ways to talk about and show the child objects and activities in their environment and encourage caregivers to respond to the child’s vocalizations and actions. CHWs created a happy atmosphere and modeled giving praise and showing love throughout the visit.
Telephone calls: Followed a similar sequence, guided by a script. For each activity, the CHW asked the caregiver to turn to the relevant page in the parent handbook and described the new activity. The CHW then asked the caregiver to try it with the child and to explain what they did. The CHW praised the efforts and offered further description and explanation where necessary.
Both contacts ended with a recap of activities and encouragement to continue activities daily.

**Training for CHWs and Nurses**

CHWs and nurses received training in 3-d workshops in the blended delivery method, covering conduct of home visits and telephone calls and review of intervention methods. In all, 76% of CHWs had previously received full 10-d training for home visits. CHWs were provided with the curriculum (comprising activities from the home visit curriculum and parent handbook) and visit record books.

**Supervision**

Nurses were asked to review visits monthly with CHWs individually, provide supportive feedback, and sign the CHW record books. They were also asked to discuss the program at routinely held group meetings and conduct observations of visits using a provided 15-item checklist. Supervision was reviewed with the nurses at the 3-d workshops.

Abbreviation: CHW, community health worker.

### Staff Training

Prior to initial implementation in 2019, the MOHW organized training of trainers workshops for nurses and health education officers. Workshops were conducted by the research team and covered training in the program and how to deliver the training and supervision. CHWs were trained in delivery of home visits in 10-day workshops conducted by the MOHW trainers. A member of the research team (S.M.C.) attended 1 day of the workshops and provided feedback to the MOHW trainers.

Due to staff turnover after initial training, nurses identified additional CHWs to deliver the intervention along with CHWs who participated previously. In all, 71 of 93 CHWs (76.3%) had received the 10-day training. Given the time since initial training (3 years) and staff turnover, 3-day workshops were conducted by the research team for nurses and CHWs from May to June 2022 (Box).

### Randomization and Implementation Procedure

Training and participant recruitment were done in parishes sequentially, with implementation beginning when recruitment in a parish was complete. For each CHW, a random order for participant group assignment was generated by an independent statistician and kept by a single member of the research team (S.M.C.). Following baseline interviews, participants were given the next available group assignment. CHWs were informed of the assignments and began the intervention with families assigned to the intervention group. Text messages for intervention families began 1 week later. Enrollment began in July 2022, and intervention delivery started on a phased basis from September 2022 to August 2023. End line assessments were conducted after families had received the intervention for 8 months (May 2023 to April 2024).

### Measurements

Baseline information was obtained using an interviewer-administered questionnaire.^19,23^ Family data included mother’s age, educational level, and employment status; father’s age and whether he resided in the home; and home environment variables—crowding (persons per room), type of toilet, water supply, and household possessions.^23^ The mother’s attitude concerning child development and parents’ role was assessed by maternal report.^23^ The Family Care Indicators (FCI) questionnaire was used to assess activities the primary caregiver did with the child.^25^ Maternal depressive symptoms were assessed by the brief 10-item Center for Epidemiological Studies–Depression (CES-D-10) scale (score range, 0-30, with scores ≥10 indicating depression).^26^ The CES-D has been used previously in Jamaica.^23^ Child characteristics, including date of birth, sex, birth weight, and current weight and length, were obtained from children’s clinic records.

At end line, measurements of parenting attitudes, parent practices (FCI questionnaire), and maternal depressive symptoms were repeated. Child development was assessed with the Griffiths Mental Development Scales,^24,27^ which have been used in several studies in Jamaica.^23,28,29^ Three of the 5 subscales were used—hearing and speech (language), hand and eye (fine motor), and performance (cognition)—and the DQ was calculated. Four of 6 subscales from the Home Observation for Measurement of the Environment (HOME) were used to measure parenting behaviors—involvement, responsivity, acceptance, and learning materials.^23,30^ Five university graduates, blinded to study objectives and participant group assignment, conducted interviews or testing. Training was approximately 4 weeks.

Interobserver reliability was assessed using intraclass correlations between interviewers and the trainer (A.S.W.) (n = 19 interviews; *r* > 0.97 for all questionnaires) and between testers and the trainer (S.M.C.) (n = 19 tests; *r* > 0.97 for all subscales). Ongoing measurement quality remained high for interviews (n = 15) and tests (n = 20). Measurements were conducted in private rooms in health centers.

### Information on Program Costs

Cost information was collected in 2022 using invoices from suppliers and salary scales provided by the MOHW for personnel costs. Details are provided in eTable 1 in Supplement 2. We conducted a benefit-cost analysis, following prior reports,^31,32,33^ with sensitivity analysis for discount rates, wages, and treatment intervals. To estimate the present value of benefits, we used the Jamaican efficacy trial of weekly home visiting on which Reach Up is based.^28^

### Statistical Analysis

Comparisons of enrollment characteristics for the end line sample were done using *t* tests or χ^2^ analyses. Principal-component analysis of crowding, water and toilet ratings, and household possessions was used to generate a socioeconomic status (SES) factor score. Multivariate regression analyses were used to examine the treatment effect. A 2-sided *P* value less than .05 was considered to be statistically significant.

Analysis was by intention to treat. For child development outcomes, group assignment (control = 0, intervention = 1), child sex and age at assessment, and dummy variables for tester were entered. Mother’s educational level (completed secondary level or not), maternal depressive symptoms, and the SES factor score were included as covariates, as these have been related to development in prior studies.^23,34^

We used 3 variables—maternal age, maternal educational level, and household sanitation scores (sum of water and toilet ratings), which differed between participants lost to follow-up and those assessed at end line—to estimate probability of loss and calculated inverse probability weights (IPWs). IPWs were used to adjust for any bias in loss to follow-up.

The same model (omitting tester dummy variables) was used for parent outcomes. We then examined whether impact varied by maternal characteristics (educational level, depressive symptoms) or child’s age and sex using interaction terms. Analyses were conducted with Stata/SE, version 16.0 (StataCorp LLC).

## Results

The total sample enrolled was 627 children (311 randomized to intervention and 316 to control; 305 [48.6%] female and 322 [51.4%] male). A total of 491 children were assessed at end line (237 [76.2%] of those in the intervention group and 254 [80.4%] of controls), a follow-up rate of 78.3% (Figure). A total of 393 mothers (62.7%) had completed secondary school. Children lost to follow-up had younger mothers, mothers with higher school grade completed, and higher sanitation scores (sum of water and toilet ratings) than those assessed at end line. There were no other significant differences. Participant characteristics on enrollment are shown in Table 1 for the total sample and end line sample. There were no statistically significant differences in characteristics or in loss to follow-up by group. Children’s mean (SD) ages at end line were 27.0 (5.8) months in the control group and 27.0 (6.1) months in the intervention group; the end line sample consisted of 237 females (48.3%) and 254 males (51.7%). A maximum of 16 contacts occurred per participant (2 per month), with a median of 7 (IQR, 2-10).

**Figure.**
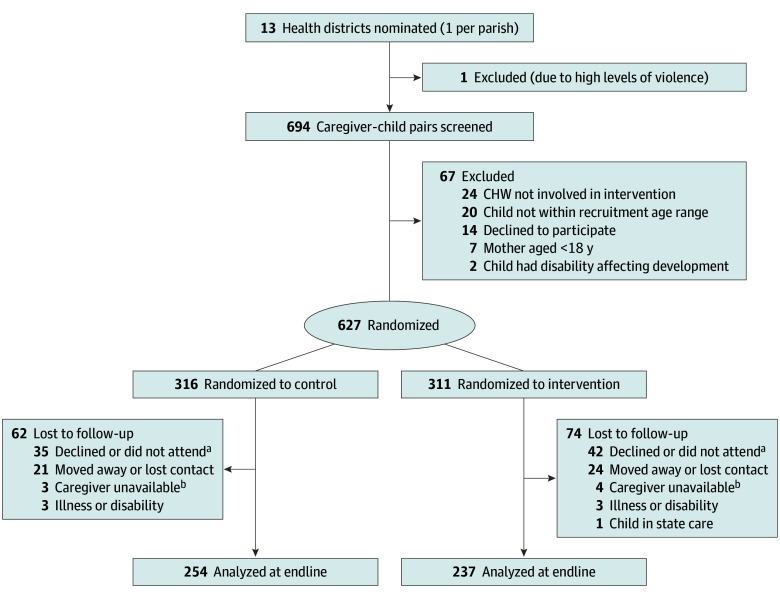
CONSORT Diagram CHW indicates community health worker. ^a^Caregiver did not attend assessment after 2 appointments. ^b^Caregiver was unavailable to attend due to work.

**Table 1.** Enrollment Characteristics by Group at Enrollment and at End Line

Characteristic	Participants^a^			
	Total sample (N = 627)		End line sample (n = 491)	
	Control (n = 316)	Intervention (n = 311)	Control (n = 254)	Intervention (n = 237)
**Children**				
Sex				
Female	144 (45.6)	161 (51.8)	117 (46.1)	120 (50.6)
Male	172 (54.4)	150 (48.2)	137 (53.9)	117 (49.4)
Birth weight, kg	3.09 (0.53)	3.06 (0.60)	3.08 (0.53)	3.07 (0.64)
Age at enrollment, mo	14.2 (5.6)	14.3 (5.7)	14.3 (5.6)	14.3 (5.7)
z Score				
Height for age	−0.29 (1.32)	−0.36 (1.32)	−0.29 (1.30)	−0.38 (1.38)
Weight for length	0.43 (1.19)	0.37 (1.14)	0.44 (1.17)	0.38 (1.14)
Mother’s first child	106 (33.5)	97 (31.2)	89 (35.0)	71 (30.0)
**Caregivers**				
Age, y	29.6 (9.0)	28.5 (8.3)	29.9 (9.3)	29.0 (8.3)
Maternal characteristics				
Highest educational grade completed	10.3 (1.3)	10.2 (1.7)	10.2 (1.4)	10.1 (1.8)
Completed secondary education	202 (63.9)	191 (61.4)	161 (63.4)	139 (58.6)
Employed	158 (50.0)	151 (48.6)	124 (48.8)	115 (48.5)
Occupation^b^				
None or unskilled	55 (17.4)	49 (15.8)	49 (19.3)	38 (16.0)
Semiskilled	135 (42.7)	139 (44.7)	106 (41.7)	104 (43.9)
Skilled or highly skilled	126 (39.9)	123 (39.5)	99 (39.0)	95 (40.1)
Depressive symptoms (CES-D-10 score)^c^	11.1 (6.6)	11.4 (6.6)	11.2 (6.7)	11.5 (6.6)
Parenting attitudes, total score^d^	30.6 (4.0)	30.7 (3.9)	30.5 (3.9)	30.7 (3.8)
FCI activities score^e^	2.9 (1.3)	2.9 (1.3)	2.9 (1.3)	2.9 (1.3)
Father lives with child	143 (45.3)	141 (45.3)	118 (46.5)	111 (46.8)
**Home environment**				
Possessions, No.^f^	7.9 (2.7)	7.7 (2.6)	8.0 (2.7)	7.6 (2.6)
Crowding, persons/room, No.	2.1 (1.3)	2.1 (1.4)	2.0 (1.2)	2.1 (1.3)
Sanitation (water and toilet ratings)^g^	8.8 (3.2)	8.5 (3.3)	8.6 (3.2)	8.4 (3.2)
SES factor score^h^	0.04 (0.99)	−0.04 (1.01)	0.04 (0.98)	−0.07 (1.00)

Abbreviations: CES-D-10, 10-item Center for Epidemiological Studies Depression Scale; FCI, Family Care Indicators questionnaire; SES, socioeconomic status.

^a^ Data are presented as number (percentage) of participants for categorical variables and mean (SD) for continuous variables.

^b^ Current or last occupation.

^c^ CES-D-10 score range, 0 to 30; scores of 10 or higher indicate depression.

^d^ Parenting attitudes were assessed by questionnaires (score range, 10-40; higher scores indicate more positive attitudes toward parenting).

^e^ Number of types of play and language activities per day (from a list of 6).

^f^ From a list of 14 items, including household appliances and vehicles (full list is in the trial protocol in Supplement 1).

^g^ Sanitation score range, 0 to 12, with higher scores indicating better sanitation.

^h^ SES factor score (possessions, crowding, and water and toilet ratings) is a continuous variable, with higher scores indicating higher SES.

Child, parent, and home environment outcomes are shown in Table 2 and Table 3. Unadjusted analyses showed fine motor and HOME scores were significantly greater in the intervention than in the control group (Table 2), and more caregivers in the intervention group made toys and praised their child when they behaved well (Table 3). No other outcomes were significantly different between the groups.

**Table 2.** Multivariate Linear Regression Analyses of Intervention Impact on Child Development and Parent and Home Environment Outcomes at End Line by Group

Outcome	Participants, mean (SD)		Unadjusted B (SE) [95% CI]^a^	*P* value	Adjusted B (SE) [95% CI]^b^	*P* value
	Control (n = 254)	Intervention (n = 237)				
**Child**						
Developmental quotient^c^	89.2 (12.3)	91.3 (14.1)	2.08 (1.20) [−0.27 to 4.45]	.08	2.31 (1.08) [0.19 to .42]	.03
Language^d^	90.6 (18.6)	92.1 (18.0)	1.53 (1.66) [−1.72 to 4.79]	.35	1.69 (1.53) [−1.33 to 4.71]	.27
Fine motor ability^e^	89.2 (13.5)	91.9 (15.5)	2.77 (1.31) [0.20 to 5.34]	.04	2.82 (1.22) [0.42 to 5.23]	.02
Cognition and problem solving^f^	87.9 (14.3)	89.8 (16.8)	1.94 (1.41) [−0.83 to 4.70]	.17	2.40 (1.29) [−0.13 to 4.93]	.06
**Parent and home environment**						
Mother’s depressive symptoms (CES-D-10 score)^g^	11.0 (6.7)	10.8 (6.8)	−0.20 (0.61) [−1.40 to 1.01]	.75	NA	NA
Parenting attitudes score^h^	31.2 (4.0)	31.8 (3.6)	0.57 (0.34) [−0.11 to 1.24]	.10	0.63 (0.33) [−0.03 to 1.28]	.06
HOME total score^i^	20.4 (3.8)	21.2 (3.5)	0.78 (0.33) [0.13 to 1.43]	.02	0.90 (0.32) [0.28 to 1.52]	.004
FCI activities score^j^	2.5 (1.4)	2.6 (1.3)	0.05 (0.12) [−0.19 to 0.28]	.68	NA	NA
Language rating^k^	7.9 (1.4)	8.0 (1.3)	0.08 (0.13) [−0.17 to 0.33]	.51	NA	NA

Abbreviations: CES-D-10, 10-item Center for Epidemiological Studies Depression Scale; FCI, Family Care Indicators questionnaire; HOME, Home Observation for Measurement of the Environment; NA, not applicable.

^a^ Unadjusted regression was used for group assignment only.

^b^ For the adjusted regression, all models included group assignment (0 = control; 1 = intervention), child age and sex, socioeconomic status factor score, maternal depressive symptoms, and whether the mother had completed secondary education. Child development regressions include dummy variables for tester. Inverse probability weights were used to adjust for any bias in loss to follow-up.

^c^ Developmental quotient score range, 60 to 140 points, with lower scores indicating delayed development.

^d^ Language score range (age 0-2 y), 60 to 158, with lower scores indicating delayed development.

^e^ Fine motor ability score range (age 0-2 y), 51 to 149, with lower scores indicating delayed development.

^f^ Cognition and problem solving score range (age 0-2 y), 51 to 149, with lower scores indicating delayed development.

^g^ CES-D-10 score range, 0 to 30; scores of 10 or higher indicate depression.

^h^ Parenting attitudes were assessed by questionnaires (score range, 10-40; higher scores indicate more positive attitudes toward parenting).

^i^ HOME score range, 0 to 30, with higher scores indicating more stimulating home environment.

^j^ Number of types of play and language activities per day (from a list of 6).

^k^ Sum of 3 ratings of mother-child verbal interaction.

**Table 3.** Multivariate Logistic Regression Analyses of Intervention Impact on Parent and Home Environment Outcomes at End Line by Group

Outcome	Participants No. (%)		Unadjusted OR (SE) [95% CI]^a^	*P* value	Adjusted OR (SE) [95% CI]^b^	*P* value
	Control (n = 252)	Intervention (n = 237)				
Praise if child behaved well most of the time	185 (73.4)	197 (83.1)	1.78 (0.40) [1.15-2.77]	.01	1.84 (0.42) [1.17-2.89]	.008
Praise if child did something that made the parent happy, No./total No. (%)	132/237 (55.7)	131/219 (59.9)	1.12 (0.20) [0.79-1.60]	.52	NA	NA
Parent made toy(s) for child						
No	175 (69.4)	133 (56.1)	1.78 (0.34) [1.23-2.58]	.002	1.71 (0.33) [1.17-2.48]	.005
Yes	77 (30.6)	104 (43.9)				
Other toys in home, No.						
0-8	78 (31.0)	78 (32.9)	0.94 (0.16) [0.68-1.31]	.73	NA	NA
9-14	82 (32.5)	74 (31.2)				
≥15	92 (36.5)	85 (35.9)				
Children’s books in home, No.						
0-1	96 (38.1)	78 (32.9)	1.22 (0.20) [0.88 to 1.69]	.24	NA	NA
2-3	77 (30.6)	76 (32.1)				
≥4	79 (31.3)	83 (35.0)				

Abbreviation: NA, not applicable.

^a^ Unadjusted regression was used for group assignment only.

^b^ For the adjusted regression, all models included group assignment (0 = control; 1 = intervention), child age and sex, socioeconomic status factor score, maternal depressive symptoms, and whether the mother had completed secondary education. Child development regressions include dummy variables for tester. Inverse probability weights were used to adjust for any bias in loss to follow-up.

Multivariate regression analyses were conducted for the primary outcomes of child development and for 4 of 10 parent outcomes where unadjusted analyses showed difference at *P* ≤ .10 (Table 2). Children’s DQ ES (0.17 SD; 95% CI, 0.01-0.33 SD), fine motor subscale score ES (0.19 SD; 95% CI, 0.03-0.36 SD), and HOME score ES (0.25 SD; 95% CI, 0.08-0.41 SD) were higher in the intervention group. There were no between-group differences in language or cognition subscale scores or in parenting attitudes. Logistic regression analyses (Table 3) showed caregivers in the intervention group were more likely to use praise when their child behaved well and to have made toys for their child.

There was no significant heterogeneity of impact by child age (above or below the median age of 26.4 months [IQR, 12.2-46.9 months]), child sex, mother’s educational level (completed secondary-level or not), or maternal depressive symptoms (CES-D-10 score of 0-9 vs 10-30). The estimated annual cost per child per year for the intervention was US $245.79 (eTable 1 in Supplement 2).

For the 8-month intervention period, the cost was US $163.86 per child. To estimate the present value of benefits, we used the Jamaican efficacy trial, which had an impact on DQ of 0.80 SD.^28^ The ES of 0.17 SD in the current trial was 21% of the former. Follow-up of the Jamaican efficacy trial participants at age 21 years found 25% higher wages for the treatment group.^35^ We multiplied the 25% impact by 0.21 to estimate the impact of the intervention, resulting in an estimated wage increase of 5.3%. We multiplied 0.053 by the average wage to estimate the yearly wage return and used a discount rate of 5% to estimate the net present value of these income gains. Assuming participants enter the labor market at age 17 years and retire at age 65 years and assuming an average annual wage in 2022 of US $4394 (estimated from the weekly minimum wage), the summed yearly gains resulted in a total discounted benefit of $2016 with a benefit-cost ratio of 12.3. Sensitivity analyses and lower and upper bounds are shown in eTable 2 in Supplement 2.

## Discussion

Blended delivery of the Reach Up program showed modest benefits to children’s development and parenting behaviors. Implementation and most costs were undertaken by the MOHW, with limited support from the research team related to costs of text messaging and telephone calls. Effect sizes for child development were within the range of other effectiveness trials of Reach Up^36,37,38^ and of other parenting programs using home visits,^10,39,40,41^ although they were lower than the average effect sizes in a meta-analysis of randomized clinical trials evaluating Reach Up^14^ that included small-scale efficacy trials,^28,29,42^ where impact is usually greater. Furthermore, in several effectiveness trials in the meta-analysis, delivery staff and supervisors were hired for the trial and trained by the research teams.^36,43,44^ In 2 trials in Bangladesh, the intervention was delivered by government health workers with large benefits for child development.^11,45^ However, health workers were trained and supervised by the research team. In the current trial, MOHW trainers conducted the main training of CHWs, and supervision was done by nurses.

No significant effect moderation was found when considering child age and sex, maternal educational level, and maternal depressive symptoms. Two meta-analyses^4,14^ suggested impact may be greater for children aged older than 12 months at the start of intervention compared with younger children. In the current study, the median age was above that cutoff. Moderation by child sex is likely to vary by context, with evidence of greater impact in girls in Kenya,^46^ whereas no moderation was observed in Colombia.^37^ Lack of moderation by maternal educational level is consistent with several studies.^14,37,46^ Although the importance of maternal mental health for children’s development is recognized,^2,47^ few studies have examined moderation of intervention impact by maternal depressive symptoms. Further studies examining this are needed.

Implementation of the program was disrupted in 2020 by the COVID-19 pandemic, leading to a gap of 3 years between the 10-day training of CHWs in 2019 and the current implementation. Staff turnover during this period resulted in 1 in 4 of the CHWs who delivered the current program having not participated in that training. The research team therefore conducted 3-day training for all CHWs and nurses prior to the delivery of the blended program, which may not have been sufficient for those without prior training.

CHWs missed contacts because of caregiver unavailability, violence in the community, or conflicting duties such as immunization drives in schools, and in some cases, contacts were missed because the CHWs lacked motivation to conduct the visits. In several centers, monthly supervision by nurses was either not done or done inconsistently, largely because of other work commitments. Similar challenges with staff attrition, staff overwork, and provision of supervision have been reported in other evaluations of home visiting programs integrated in government systems.^48,49,50,51,52^ This study reinforces the importance of attention to staff workload and motivation in future scaling of ECD interventions.

The annual cost per child of the intervention was approximately one-third of that in the original Jamaica efficacy trial^53^ and lower than the costs for weekly home visits in Colombia, Peru, and China.^37,48,53^ The benefit-cost ratio of 12.3 in this study is higher than that of 5.4 reported in Peru^48^ and similar to estimates reported by Karoly^31^ and the higher end of the range (5-11.7) in Nicaragua.^41^ Unlike most other estimates of long-term benefits of ECD interventions,^54^ ours were based on findings for the intervention in the same country (Jamaica).

### Limitations

This study has limitations. Evaluation was conducted after 8 months with 2 contacts per month, giving a maximum of 16 contacts with a median of 7 (IQR, 2-10) delivered. This is relatively short compared with prior studies that have generally lasted 1 to 2 years.^14^ The intervention impact on the HOME score and on use of praise and making toys indicated gains in parenting behaviors; with longer duration, these gains may have contributed to larger impact on child development.

The MOHW trained all CHWs in the health districts so that the program could be sustained after evaluation and centers would have capacity to provide the intervention to the waiting list group. Individual randomization was therefore used rather than randomization by center. This introduced the possibility of spillover if CHWs provided elements of the intervention to control families. This is unlikely, as the CHWs did not routinely visit these families. Nonetheless, it is possible that some spillover may have occurred.

Based on a study by Barnett and Masse,^32^ we assumed constant returns in our benefit-cost analysis. Furthermore, the earlier trial^28^ was highly controlled, and long-term impact would likely be lower in most at-scale settings.

## Conclusions

This randomized clinical trial of blended delivery of a parenting program by a government primary health care system found benefits to children’s DQ and fine motor ability and the quality of their home environment. This suggests that combining remote and in-person visits could be used to expand coverage of ECD programs. Through our ongoing collaboration with Jamaica’s MOHW, a 5-year plan for ECD has been developed that includes phased scaling of this parenting program. This reinforces the value of sustained partnerships between researchers and policymakers for scaling ECD programs.

## Notes

Supplement 1.
Trial Protocol

Supplement 2. **eTable 1.** Estimated Annual Intervention Costs in USD Using 2022 World Bank Conversion Rate for Jamaican Dollars **eTable 2.** Benefit-Cost Ratio—Sensitivity Analysis
eReferences

Supplement 3.
Data Sharing Statement

## References

[1] World Health Organization, United Nations Children’s Fund, World Bank Group. Nurturing Care for Early Childhood Development: A Framework for Helping Children Survive and Thrive to Transform Health and Human Potential. World Health Organization; 2018. Accessed December 22, 2025. https://www.who.int/publications/i/item/9789241514064

[2] World Health Organization. Improving Early Childhood Development: WHO Guideline. World Health Organization; 2020. Accessed December 22, 2025. https://www.who.int/publications/i/item/97892400020986 32200595

[3] Britto PR, Lye SJ, Proulx K, ; Early Childhood Development Interventions Review Group, for the Lancet Early Childhood Development Series Steering Committee. Nurturing care: promoting early childhood development. Lancet. 2017;389(10064):91-102. doi:10.1016/S0140-6736(16)31390-3 27717615

[4] Jeong J, Franchett EE, Ramos de Oliveira CV, Rehmani K, Yousafzai AK. Parenting interventions to promote early child development in the first three years of life: a global systematic review and meta-analysis. PLoS Med. 2021;18(5):e1003602. doi:10.1371/journal.pmed.1003602 33970913 PMC8109838

[5] Ahun MN, Ali NB, Hentschel E, Jeong J, Franchett E, Yousafzai AK. A meta-analytic review of the implementation characteristics in parenting interventions to promote early child development. Ann N Y Acad Sci. 2024;1533(1):99-144. doi:10.1111/nyas.15110 38354095

[6] Nores M, Vazquez C, Gustafsson-Wright E, . The cost of not investing in the next 1000 days: implications for policy and practice. Lancet. 2024;404(10467):2117-2130. doi:10.1016/S0140-6736(24)01390-4 39571590

[7] Powell C, Baker-Henningham H, Walker S, Gernay J, Grantham-McGregor S. Feasibility of integrating early stimulation into primary care for undernourished Jamaican children: cluster randomised controlled trial. BMJ. 2004;329(7457):89. doi:10.1136/bmj.38132.503472.7C 15217841 PMC449816

[8] Yousafzai AK, Rasheed MA, Rizvi A, Armstrong R, Bhutta ZA. Effect of integrated responsive stimulation and nutrition interventions in the Lady Health Worker programme in Pakistan on child development, growth, and health outcomes: a cluster-randomised factorial effectiveness trial. Lancet. 2014;384(9950):1282-1293. doi:10.1016/S0140-6736(14)60455-4 24947106

[9] Fernald LC, Kagawa RM, Knauer HA, Schnaas L, Guerra AG, Neufeld LM. Promoting child development through group-based parent support within a cash transfer program: experimental effects on children’s outcomes. Dev Psychol. 2017;53(2):222-236. doi:10.1037/dev0000185 27748620

[10] Jensen SK, Placencio-Castro M, Murray SM, . Effect of a home-visiting parenting program to promote early childhood development and prevent violence: a cluster-randomized trial in Rwanda. BMJ Glob Health. 2021;6(1):e003508. doi:10.1136/bmjgh-2020-003508 33514591 PMC7849888

[11] Mehrin SF, Hasan MI, Tofail F, . Integrating a group-based, early childhood parenting intervention into primary health care services in rural Bangladesh: a cluster-randomized controlled trial. Front Pediatr. 2022;10:886542. doi:10.3389/fped.2022.886542 35783319 PMC9245711

[12] Hossain SJ, Roy BR, Sujon HM, . Effects of integrated psychosocial stimulation (PS) and unconditional cash transfer (UCT) on children’s development in rural Bangladesh: a cluster randomized controlled trial. Soc Sci Med. 2022;293:114657. doi:10.1016/j.socscimed.2021.114657 34942577

[13] Reach Up: an early childhood parenting programme. Caribbean Institute for Health Research. 2021. Accessed December 22, 2025. https://reachupandlearn.com/

[14] Jervis P, Coore-Hall J, Pitchik HO, . The Reach Up parenting program, child development, and maternal depression: a meta-analysis. Pediatrics. 2023;151(suppl 2):e2023060221D. doi:10.1542/peds.2023-060221D 37125892

[15] Rubio-Codina M, Boo FL. What have we learned from the design and delivery of remote and hybrid early childhood development services during the pandemic? Inter-American Bank discussion paper IDB-DP-00963. 2022.

[16] Lopez Boo F, Tome R, Rubio-Codina M, Daga G. *Social Protection Sector Framework*. Inter-American Development Bank; 2024. Accessed December 22, 2025. https://www.iadb.org/en/who-we-are/topics/social-protection/sector-framework-social-protection

[17] Carta JJ, Lefever JB, Bigelow K, Borkowski J, Warren SF. Randomized trial of a cellular phone-enhanced home visitation parenting intervention. Pediatrics. 2013;132(suppl 2)(suppl 2):S167-S173. doi:10.1542/peds.2013-1021Q 24187120 PMC4258827

[18] York BN, Loeb S, Doss C. One step at a time: the effects of an early literacy text-messaging program for parents of preschoolers. J Hum Resour. 2019;54(3):537-566. doi:10.3368/jhr.54.3.0517-8756R

[19] Smith JA, Chang SM, Brentani A, . A remote parenting program and parent and staff perspectives: a randomized trial. Pediatrics. 2023;151(suppl 2):e2023060221F. doi:10.1542/peds.2023-060221F 37125881

[20] Balsa A, Bloomfield J, Cid A. The conceptual replication of Crianza Positiva e-messaging program during the COVID-19 pandemic: too much or too little information? J Fam Econ Issues. 2025;46(4):1260-1274. doi:10.1007/s10834-024-09975-7

[21] Rafla J, Schwartz K, Yoshikawa H, . Cluster randomized controlled trial of a phone-based caregiver support and parenting program for Syrian and Jordanian families with young children. Early Child Res Q. 2024;69:141-153. doi:10.1016/j.ecresq.2024.07.004

[22] Chang-Lopez SM, Walker S, Grantham-McGregor SM, . *Manual for Parents: Early Stimulation Activities for Children up to 3 Years of Age*. Inter-American Development Bank; 2020. Accessed December 22, 2025. https://publications.iadb.org/es/manual-para-padres-actividades-de-estimulacion-temparana-para-ninos-de-hasta-3-anos-de-edad

[23] Chang SM, Grantham-McGregor SM, Powell CA, . Integrating a parenting intervention with routine primary health care: a cluster randomized trial. Pediatrics. 2015;136(2):272-280. doi:10.1542/peds.2015-0119 26148947

[24] Huntley M. The Griffiths Mental Development Scales: From Birth to 2 Years. Association for Research in Infant & Child Development; 1996.

[25] Hamadani JD, Tofail F, Hilaly A, Huda SN, Engle P, Grantham-McGregor SM. Use of family care indicators and their relationship with child development in Bangladesh. J Health Popul Nutr. 2010;28(1):23-33. doi:10.3329/jhpn.v28i1.4520 20214083 PMC2975843

[26] Andresen EM, Malmgren JA, Carter WB, Patrick DL. Screening for depression in well older adults: evaluation of a short form of the CES-D (Center for Epidemiologic Studies Depression Scale). Am J Prev Med. 1994;10(2):77-84. doi:10.1016/S0749-3797(18)30622-6 8037935

[27] Luiz D, Barnard A, Knoesen N, . Griffiths Mental Development Scales—Extended Revised: Two to Eight Years: Administration Manual. Hogrefe; 2006.

[28] Grantham-McGregor SM, Powell CA, Walker SP, Himes JH. Nutritional supplementation, psychosocial stimulation, and mental development of stunted children: the Jamaican Study. Lancet. 1991;338(8758):1-5. doi:10.1016/0140-6736(91)90001-6 1676083

[29] Walker SP, Chang SM, Powell CA, Grantham-McGregor SM. Psychosocial intervention improves the development of term low-birth-weight infants. J Nutr. 2004;134(6):1417-1423. doi:10.1093/jn/134.6.1417 15173406

[30] Caldwell BM, Bradley RH. HOME (Home Observation and Measurement of the Environment) Inventory Administration Manual. University of Arkansas; 2003.

[31] Karoly LA. Toward standardization of benefit-cost analysis of early childhood interventions. J Benefit Cost Anal. 2012;3(1):1-45. doi:10.1515/2152-2812.1085

[32] Barnett WS, Masse LN. Comparative benefit–cost analysis of the Abecedarian program and its policy implications. Econ Educ Rev. 2007;26(1):113-125. doi:10.1016/j.econedurev.2005.10.007

[33] Reynolds AJ, Temple JA, White BA, Ou SR, Robertson DL. Age 26 cost-benefit analysis of the Child-Parent Center early education program. Child Dev. 2011;82(1):379-404. doi:10.1111/j.1467-8624.2010.01563.x 21291448 PMC3817956

[34] Smith JA, Powell CA, Chang SM, Ganga E, Tanyanyiwa H, Walker SP. A cluster randomised controlled trial of an early childhood parenting programme delivered through early childhood education centres in rural Zimbabwe. Child Care Health Dev. 2024;50(1):e13189. doi:10.1111/cch.13189 37882173

[35] Gertler P, Heckman J, Pinto R, . Labor market returns to an early childhood stimulation intervention in Jamaica. Science. 2014;344(6187):998-1001. doi:10.1126/science.1251178 24876490 PMC4574862

[36] Attanasio OP, Fernández C, Fitzsimons EO, Grantham-McGregor SM, Meghir C, Rubio-Codina M. Using the infrastructure of a conditional cash transfer program to deliver a scalable integrated early child development program in Colombia: cluster randomized controlled trial. BMJ. 2014;349:g5785. doi:10.1136/bmj.g5785 25266222 PMC4179481

[37] Attanasio O, Baker-Henningham H, Bernal R, Meghir C, Pineda D, Rubio-Codina M. Early stimulation and nutrition: the impacts of a scalable intervention. J Eur Econ Assoc. 2022;20(4):1395-1432. doi:10.1093/jeea/jvac005 35965610 PMC9372035

[38] Galasso E, Weber AM, Stewart CP, Ratsifandrihamanana L, Fernald LCH. Effects of nutritional supplementation and home visiting on growth and development in young children in Madagascar: a cluster-randomised controlled trial. Lancet Glob Health. 2019;7(9):e1257-e1268. doi:10.1016/S2214-109X(19)30317-1 31402006

[39] Luo R, Emmers D, Warrinnier N, Rozelle S, Sylvia S. Using community health workers to deliver a scalable integrated parenting program in rural China: a cluster-randomized controlled trial. Soc Sci Med. 2019;239:112545. doi:10.1016/j.socscimed.2019.112545 31568997 PMC7249221

[40] Sudfeld CR, Bliznashka L, Ashery G, Yousafzai AK, Masanja H. Effect of a home-based health, nutrition and responsive stimulation intervention and conditional cash transfers on child development and growth: a cluster-randomised controlled trial in Tanzania. BMJ Glob Health. 2021;6(4):e005086. doi:10.1136/bmjgh-2021-005086 33906847 PMC8088247

[41] Lopez Boo F, Leer J, Kamei A. The importance of quality for scaling up early childhood development services: experimental evidence from Nicaragua. World Bank Econ Rev. Published online December 8, 2025. doi:10.1093/wber/lhaf036

[42] Hamadani JD, Huda SN, Khatun F, Grantham-McGregor SM. Psychosocial stimulation improves the development of undernourished children in rural Bangladesh. J Nutr. 2006;136(10):2645-2652. doi:10.1093/jn/136.10.2645 16988140

[43] Grantham-McGregor S, Adya A, Attanasio O, . Group sessions or home visits for early childhood development in India: a cluster RCT. Pediatrics. 2020;146(6):e2020002725. doi:10.1542/peds.2020-002725 33148771 PMC7786825

[44] Heckman JJ, Liu B, Lu M, Zhou J. The impacts of a prototypical home visiting program on child skills. National Bureau of Economic Research working paper 27356. June 2020. Accessed December 22, 2025. https://www.nber.org/papers/w27356

[45] Hamadani JD, Mehrin SF, Tofail F, . Integrating an early childhood development programme into Bangladeshi primary health-care services: an open-label, cluster-randomised controlled trial. Lancet Glob Health. 2019;7(3):e366-e375. doi:10.1016/S2214-109X(18)30535-7 30784637

[46] Luoto JE, Lopez Garcia I, Aboud FE, . Group-based parenting interventions to promote child development in rural Kenya: a multi-arm, cluster-randomised community effectiveness trial. Lancet Glob Health. 2021;9(3):e309-e319. doi:10.1016/S2214-109X(20)30469-1 33341153 PMC8054650

[47] Draper CE, Yousafzai AK, McCoy DC, . The next 1000 days: building on early investments for the health and development of young children. Lancet. 2024;404(10467):2094-2116. doi:10.1016/S0140-6736(24)01389-8 39571589 PMC7617681

[48] Araujo MC, Dormal M, Grantham-McGregor S, Lazarte F, Rubio-Codina M, Schady N. Home visiting at scale and child development. Journal of Public Economics Plus. 2021;2:100003. doi:10.1016/j.pubecp.2021.100003

[49] Hill Z, Zafar S, Soremekun S, . Can home visits for early child development be implemented with sufficient coverage and quality at scale? evidence from the SPRING program in India and Pakistan. Front Nutr. 2023;10:1152548. doi:10.3389/fnut.2023.1152548 37404854 PMC10315833

[50] Aboud F, Choden K, Tusiimi M, . A tale of two programs for parents of young children: independently-conducted case studies of workforce contributions to scale in Bhutan and Rwanda. Children (Basel). 2023;10(8):1413. doi:10.3390/children10081413 37628412 PMC10453503

[51] Santos IS, Munhoz TN, Barcelos RS, . Evaluation of the Happy Child Program: a randomized study in 30 Brazilian municipalities. Cien Saude Colet. 2022;27(12):4341-4363. doi:10.1590/1413-812320222712.13472022 36383848

[52] Lopez Boo F, de la Paz Ferro M, Carneiro P. Effects of integrating early childhood with health services: experimental evidence from the Cresça com Seu Filho home-visiting program. J Hum Cap. 2025;19(1). doi:10.1086/734420

[53] Zhou J, Heckman JJ, Liu B, Lu M, Chang SM, Grantham-McGregor S. Comparing China REACH and the Jamaica home visiting program. Pediatrics. 2023;151(suppl 2):e2023060221I. doi:10.1542/peds.2023-060221I 37125889 PMC10802190

[54] Hurley KM, Yousafzai AK, Lopez-Boo F. Early child development and nutrition: a review of the benefits and challenges of implementing integrated interventions. Adv Nutr. 2016;7(2):357-363. doi:10.3945/an.115.010363 26980819 PMC4785470

