# Supplementary material for: Blended Delivery of a Primary Care Parenting Program for Child Development: A Randomized Clinical Trial
Source: JAMA Netw Open. 2026 Feb 3;9(2):e2556024. doi: 10.1001/jamanetworkopen.2025.56024 (PMC12869336; doi:10.1001/jamanetworkopen.2025.56024)
Supplement: Supplement 1. — Trial Protocol [file jamanetwopen-e2556024-s001.pdf]

Supplemental materials

1. Trial Protocol - Proposal for The University of the West Indies Ethics Committee

Trial Protocol.

Proposal for The University of the West Indies Ethics Committee

Title: Reach Up early childhood parenting programme: Evaluation of Implementation and Benefits in Jamaica (ECP 187, 17/18)

Principal Investigators: Susan Walker, Susan Chang-Lopez, Epidemiology Research Unit, Caribbean Institute for Health Research, The University of the West Indies

Co-investigators: Joanne Smith, Amika Wright, Christine Powell, Epidemiology Research Unit, Caribbean Institute for Health Research, The University of the West Indies; Julia Rowe-Porter, Kemisha Shaw-Kelly and Melody Ennis, Family Health Unit, The Ministry of Health, Jamaica.

Summary of Study

Significance and justification: The 2018 WHO Nurturing Care Framework calls on governments to provide interventions for families to support responsive caregiving and early learning for children 0-3 years. The Ministry of Health and Wellness plans to implement the Reach Up early childhood parenting programme through health centres in Jamaica. Initial evaluation of the implementation utilizing remote delivery methods showed benefits to parent practices and was acceptable to the parents and health staff. In this phase of implementation, we will assess the impact on child development and parenting practices when implemented at larger scale.

Study Design: The intervention will be implemented using a blended approach (home visits and phone calls) in one health district per parish over an 8-month period. Caregivers will receive a parent manual of play activities and a few play materials. Caregivers will also receive weekly text messages to encourage them to continue the activities with the child. Impact evaluations will be conducted in all parishes, within the health districts participating in the study. Trained community health aides (CHAs) will conduct fortnightly intervention contacts (one home visit and one phone call alternately). Each CHA will be assigned 8 families, with 4 randomly assigned to intervention and 4 to the waitlist group. With approximately 8 CHAs per district, sample size will be 416 in intervention and 416 in comparison (wait list) groups.

Baseline questionnaires will be administered to obtain information on family and maternal characteristics. Impact evaluation, including assessment of child development and parenting behaviours will be conducted after the 8-month intervention period.

Benefits and Risks: There are no specific benefits to participants. The risks to participants are minimal.

Confidentiality: All questionnaires and child assessments will be conducted in private with the child and parent. Participants will be identified by their ID number only on all study documents.

The best interest of the participants will be respected at all times, and they will be provided maximum protection.

<u>Introduction and Justification</u>

Target 4.2 of the Sustainable Development Goals calls for all children to have access to quality early childhood development and care by 2030, and the recent child development Lancet series highlights the need for early child development interventions to be integrated into government services [1]. At the World Health Assembly, in May 2018 the WHO launched the Nurturing Care Framework that places responsive caregiving and opportunities to learn for children 0-3 years as critical components of care for young children and calls on government to provide necessary support and services for families who are the primary providers of care for young children.

The Jamaican early childhood home visiting (JHV) intervention is based on strengthening the capacity of mothers and other caregivers to promote optimal development of their children, through responsive interactions and play activities. The intervention has substantial benefits for child development and has been shown to be feasible for implementation through health services [2, 3]. The intervention is delivered through home visits by community workers and has been implemented in several countries with consistent benefits for children's cognition and language development [4]. In one longitudinal follow up substantial long- term benefits to cognition, education, mental health and income have been found at 17 and 22 years of age [5-7]. This work has been highly influential in policy in global child development.

The JHV has formed the basis for the Reach Up early childhood parenting programme. Reach Up was developed in order to increase capacity for implementation of early childhood parenting programmes in other countries and for larger numbers of families. Programme content includes a weekly and fortnightly curriculum, training manuals for supervisors and visitors including videos, and manuals on toy making, implementation planning and adaptation. The programme has now been implemented in several countries including Bangladesh [8, 9], India [10], Zimbabwe, China, Colombia [11], Brazil, Bolivia, and Peru [12]. Reach Up has been shown to be well accepted and feasible [13].

The Jamaica Ministry of Health and Wellness (MOHW) plans to implement Reach Up in all parishes in Jamaica. Implementation will be through a phased roll out beginning in one health district per parish. The intervention will be implemented on a fortnightly basis and will be conducted through the health centres in each district. We propose to use this phase of implementation to evaluate the impact of the intervention on child development and parenting when implemented at larger scale.

Research Questions

Impact evaluation: What are the benefits to child development and behaviour when the Reach Up programme is delivered through government primary health care services in Jamaica?

Methods

Selection of districts and sample

Each Parish health team, led by the Medical Officer of Health, nominated the health district within the parish for the initial implementation phase (phase 1) and two nurses to be trained in the intervention. Initial impact and implementation evaluations were conducted in six parishes that began home visits before these were suspended in March 2020. Remote delivery was conducted in these parishes beginning in September 2020 and evaluated from July to October 2021. Feedback from health staff and families suggested the use of a blended intervention with home visits and phone calls. In this phase of the study the impact of the blended intervention on child development and parent practices will be evaluated.

The intervention will focus on children aged 6-24 months who meet at least one of the following criteria: families where the child or mother is registered with the Programme of Advancement through Health and Education (PATH) or the family resides in a low income setting. Children identified and whose mother provides consent to participate will be randomly assigned to either receive the intervention now or after 8 months (wait list group). Children assigned to the 'wait list' group will continue to receive usual care from the health centres.

Within each district the intervention will be implemented in 1-3 health centres that will comprise those supervised by the nurses trained. All community health aides (CHAs) assigned to these health centres will be trained to deliver the intervention, approximately 8 CHAs per district. So as to enable the CHAs to conduct the fortnightly intervention contacts (home visits and phone calls) together with their usual duties, each CHA will be assigned 8 families with 4 randomly assigned to intervention and 4 to the waitlist group. We therefore estimate 32 children in the intervention group per district and another 32 children per district in the wait list group, with a total of 416 per group across all parishes.

Mother-child pairs will be excluded if:

- Child is in Daycare or has no consistent caregiver
- The baby has major disability likely to affect development

Sample size calculation: From prior work we estimate an effect size for the primary outcome of child developmental quotient of 0.3SD (3.6 points) and SD of 12 points. For 80% power and significance level of $p<0.05$, this gives a sample size per group of 178. The actual proposed sample per group of 416 allows for possible lower recruitment levels in some districts and 10% loss to follow-up at endline. It will also allow for analysis of moderators of impact as indicated in the analysis section.
We will obtain baseline information on maternal, family and child characteristics to demonstrate equivalence among intervention arms and enable us to control for any possible

confounders at post-test. The blended intervention will continue for 8 months, and post intervention measurements will then be conducted.

Description of intervention

The blended intervention comprises fortnightly contacts with home visits and phone calls, conducted alternately, that will be delivered by CHAs for 8 months. Caregivers will receive a parent manual with play activities and a few materials. They will also receive weekly text messages that will provide encouragement to continue doing the activities with their child. The intervention works through parents by building a positive relationship to support them in strengthening skills to promote child development. The aims are to build mothers' skills, self-esteem and enjoyment in helping her child learn, through interactive play and responsive interactions. The intervention uses a structured curriculum with activities arranged in order of difficulty; children usually move on to the next set of activities with each contact.

The contacts begin with some time catching up with how the family has been and how the mother and child have progressed with the activities from the last visit. The CHA then engages the mother and child in a play session. In home visits new activities are introduced through an interactive approach: observing what the child does, demonstrating and describing the activity to mother and child, helping the child with the activity, encouraging mother and child to practise, giving positive feedback and celebrating success. Mothers are encouraged to respond to their child's vocalisations and actions. CHAs demonstrate ways to talk about and show her child objects and activities in their environment. The visitors promote giving praise, celebrating the child's achievements and efforts, and showing love throughout the visit. The visit ends with a review of activities to continue during the next two weeks and encouragement to continue the activities and to try and include them in daily routines. A similar approach is used in the phone-calls guided by a script. In this case the CHA asks the mother to try the activity with the child and to let her know what the child does. Further description and explanation are given where necessary.

CHAs will receive supervised visits or observations of the phone calls to provide supportive feedback and address any issues during the intervention period. Supervisors will use an evaluation checklist to assess the quality of the visit and the phone calls (contacts). The quality of the contact includes assessment of the interaction between the home visitor, mother and child; the atmosphere of the contact and the preparation of the home visitor for contact. Supervision will be conducted by nurses, health education officers and nutritionists.

Implementation plan/framework

The blended intervention will be implemented in one health district per parish. Training and recruitment will be done in parishes sequentially with implementation beginning as soon as recruitment in that parish is complete. Nurses from the health centres will be given training as intervention supervisors. The CHAs will also receive training in the Reach Up blended intervention and following random assignment by an independent statistician CHAs will be assigned their families by the supervisors.

Staff Training

The MOHW trained health staff including nurses, health education officers and nutritionists to supervise the CHAs and support initial programme delivery. Training workshops were conducted in phases with the nurses (Public Health Nurses and Midwives), nutritionists and health education officers being trained first. These training workshops were conducted by the UWI Reach Up team (Drs Christine Powell, Susan Chang-Lopez and Joanne Smith). The nurses, nutritionists and health education officers also received specific training on supervision and how to use the checklist provided. The CHAs were trained in separate workshops conducted by the nurses, nutritionists and health educators who were previously trained with support from the Reach Up team.

For the blended intervention delivery, 2-day training workshops will be conducted by the UWI team with the health staff (nurses and CHAs). The staff will be trained on how to conduct the phone calls and home visits using a new combined curriculum and several of the home visit intervention methodologies will also be reviewed. Nurses, health educators and nutritionists will also be trained on how to use the Observation checklist when they supervise the phone calls and visits.

Impact evaluation

Baseline measurements will be collected on enrolment to the study to obtain information on family, maternal characteristics, mother's attitudes on child development and parent practices. Impact evaluation will be conducted at the end of the 8-month intervention period.

Measurements

Baseline - An interviewer administered questionnaire will be used to obtain information from the mother on the following (Appendix 1):

Maternal and family data: including mother's age and education, employment, family structure, standard of housing (crowding- person/room, type of toilet, water supply and household possessions). Information on father's age and presence in the home will also be obtained by maternal report. We will use a questionnaire previously used in Jamaica [14].

Attitudes on child development: mother's attitude concerning child development and her role in promoting child development, using a questionnaire previously used in Jamaica [14].

Parent Practices: questions from the Family Care Indicators questionnaire will be used to assess the activities the mother or principal caretaker does with the child [15]. The FCI has been modified for use in Jamaica and was used in the evaluation of the remote delivery intervention.

Maternal depressive symptoms: measures will be obtained by interview using the Brief Center for Epidemiological Studies-Depression (CES-D-10) scale[16]. The CES-D has been used previously in Jamaica [14].

Child: Date of birth and birth weight, current length and weight, will be obtained from the children's clinic records/ child passport.

End of intervention: The following sections of the questionnaire will be repeated – Attitudes on child development, parent practices and maternal depressive symptoms. Child development will be assessed with the Griffith Scales of Development [17] which have been used in several studies in Jamaica.

Checklist, observations

Evaluation Checklist: Supervisors will use an evaluation checklist to assess the quality of the visit or call. The quality of the visit or call includes assessment of the interaction between the home visitor, mother and child; the atmosphere of the visit and the preparation of the home visitor for the visit [18]. This checklist was developed previously in Jamaica and has also been used to monitor quality in other countries [18]. **(Appendix 1)**

Ethical Issues

Impact Evaluation

Recruitment

The participants will be recruited from health centres in all parishes in Jamaica. Parents and children in the control group will continue to receive usual care and will receive the intervention at the end of this phase (8 months later). The evaluation sampling will ensure that there is no bias in selection of mothers to participate in the evaluation. The participants will be selected only because of the intervention under evaluation and not because of easy availability, diminished autonomy or social bias.

Informed Consent

Interviewers will obtain informed consent from each participant before administering the questionnaire. Participants will be informed about the study and advised that participation is voluntary and they would be able to withdraw at any time. We would also inform them that all information collected will be kept confidential. **(Appendix 2)**

Confidentiality

Questionnaires will not include the names of the mother or child to maintain confidentiality. All completed questionnaires will be kept safely in a locked file cabinet and access will be limited to research staff only. Locator information with participant names and addresses will be kept separate from the study data.

Justice

Subjects will be selected only because they attend the health centres or have certain demographic characteristics that are relevant to the objectives of this study and not because of easy availability, diminished autonomy or social bias.

Benefits

There are no direct benefits to participants, however information collected will be used to inform interventions and policy for child development in Jamaica, particularly the continued roll out of the Reach Up programme.

Risks

There are minimal risks associated with the administration of the questionnaire or conducting the measurements.

Data Analysis Plan:

Impact evaluation

Primary analyses for the impact evaluation will be by intention to treat. We will compare the groups on baseline measurements and will control for any measures which differ among the groups at endline in multivariate analyses of outcome.

Following the primary regression analyses we will investigate whether impact varies by maternal characteristics (education, depressive symptoms) or child characteristics (age on enrolment). We will also investigate the mechanism for impact to determine if change in parent practices mediates the impact on child outcomes.

References

1. Richter, L.M., et al., *Investing in the foundation of sustainable development: pathways to scale up for early childhood development.* Lancet, 2017. **389**(10064): p. 103-118.
2. Grantham-McGregor, S.M., et al., *Nutritional supplementation, psychosocial stimulation, and mental development of stunted children: the Jamaican Study.* Lancet, 1991. **338**(8758): p. 1-5.
3. Powell, C., et al., *Feasibility of integrating early stimulation into primary care for undernourished Jamaican children: cluster randomised controlled trial.* BMJ, 2004. **329**(7457): p. 89.
4. Gratham-McGregor, S. and J.A. Smith, *Extending The Jamaican Early Childhood Development Intervention.* J Appl Res Child Informing Policy Child Risk 2016. **7**(2).
5. Walker, S.P., et al., *Effects of early childhood psychosocial stimulation and nutritional supplementation on cognition and education in growth-stunted Jamaican children: prospective cohort study.* Lancet, 2005. **366**(9499): p. 1804-7.
6. Walker, S.P., et al., *Early childhood stimulation benefits adult competence and reduces violent behavior.* Pediatrics, 2011. **127**(5): p. 849-57.
7. Gertler, P., et al., *Labor market returns to an early childhood stimulation intervention in Jamaica.* Science, 2014. **344**(6187): p. 998-1001.
8. Hamadani, J.D., et al., *Psychosocial stimulation improves the development of undernourished children in rural Bangladesh.* J Nutr, 2006. **136**(10): p. 2645-52.
9. Nahar, B., et al., *Effects of a community-based approach of food and psychosocial stimulation on growth and development of severely malnourished children in Bangladesh: a randomised trial.* Eur J Clin Nutr, 2012. **66**(6): p. 701-9.
10. Andrew, A., et al., *Medium-Term Impacts of a Scalable Early Child Development Intervention to Increase Psychosocial Stimulation in the Home: A Two-Year Follow-Up of a Cluster Randomised Controlled Trial in Colombia.* Plos Medicine (in press), 2018.
11. Attanasio, O.P., et al., *Using the infrastructure of a conditional cash transfer program to deliver a scalable integrated early child development program in Colombia: cluster randomized controlled trial.* BMJ, 2014. **349**: p. g5785.
12. Araujo, M.C., et al., *Home visiting at scale: The evaluation of Peru's Cuna Mas Program.*, in *The Early Years: Child Well-Being and the Role of Public Policy*. 2016, The British Academy: London, England.
13. Smith, J.A., et al., *Implementation of Reach Up early childhood parenting program: acceptability, appropriateness, and feasibility in Brazil and Zimbabwe.* Ann N Y Acad Sci, 2018. **1419**(1): p. 120-140.
14. Chang, S.M., et al., *Integrating a Parenting Intervention With Routine Primary Health Care: A Cluster Randomized Trial.* Pediatrics, 2015. **136**(2): p. 272-80.
15. Hamadani, J.D., et al., *Use of family care indicators and their relationship with child development in Bangladesh.* Journal of health, population, and nutrition, 2010. **28**(1): p. 23.
16. Eaton, W., C. Muntaner, and C. Smith, *Centre for Epidemiologic Studies Depression Scale–Revised (CESD–R).* Innovations in clinical practice: A source book, 2001: p. 295-7.
17. Luiz, D., et al., *Griffiths Mental Development Scales - Extended Revised (GMDS-ER)*. Association for Research in Infant and Child Development, ed. ARICD. 2006, Oxford, UK.: Hogrefe - The Test Agency.
18. Leer, J. and F. Lopez-Boo, *Assessing the quality of home visit parenting programs in Latin America and the Caribbean.* Early Child Development and Care, 2018: p. 1-14.

Appendix 1

Reach Up early childhood parenting programme:
Evaluation of Implementation and Benefits in Jamaica 2022

Interviewer ________________ Date ___________ (dd/mm/yyyy)

Health Centre ID# __________ Subject ID# ________

1. Relationship of respondent to child

Mother	_______	
Father	_______	
Relative	_______	(specify)
Non-Relative	_______	

Who are the persons who live in the home with you *(indicate relationship to study child and get ages of **all children**; probe if persons **share food <u>and</u> sleep in the home ≥ 4 nights/week**). Remember to include **respondent and child** in the list of persons.*

____

____

2. Where does the mother live?

Mother lives with child	_____	(*if yes, go to #4*)
Same community/district as child	_____	
Other community/district	_____	
Abroad	_____	
Dead/unknown	_____	
Other	_____	

3. *If mother, does not live in the home*, how often does child see his/her mother?

At least once/week	_____
At least once/month	_____
Less than once/month	_____
Mother is deceased	_____

4. Where does the father live?

Father lives with child	_____	(*if yes, go to #6*)
Same community/district as child	_____	
Other community/district	_____	
Abroad	_____	
Dead/unknown	_____	
Other	_____	

5. *If father, does not live in home,* how often does child see his/her father?

At least once/week	_____
At least once/month	_____
Less than once/month	_____
Father is deceased	_____

Caregiver's Age

6. How old are you now? ____________

Caregiver's Education

7. What was the highest grade you completed in school? __________

8. Did you complete any training after you left school? Yes _____ No _____

   If yes, what

____

Caregiver's Employment

9. Do you currently work (i.e. work for someone or self-employed)?

   Yes _____ No_____ (*if no, go to #14*)

   (If on maternity leave code Yes)

10. Type of work? ________________________________

11. Part time _____ Full time ____

    If not working,

12. Have you ever worked? Yes _____ No _____(*if no, go to #14*)

13. If yes, what was the last job you did? ____

Father's age

14. How old is father now? __________ D/K ______

Child's Birth Information

15. Is this your/the mother's first child? Yes _____ No _____

    If No, how many other children do you/ does she have, *before* this child

    ____

    (record all live births, and if a sibling has died)

Can I see the baby's child health passport? ***(Child passport, pages 12-13)***

Child passport seen? Yes _____ No _____

16. Baby's sex Male _____ Female _____

17. Baby's Birth weight (kg) _________________

18. Date of birth (day/month/year) _________________

19. Where was he/she born? (*record name of hospital/home delivery*) ______________________

Housing

20. What type of toilet do you have in your home?

Own inside flush	6	Own pit	2
Shared inside flush	5	Shared pit	1
Own outside flush	4	None	0
Shared outside flush	3		

21. What is your household's main source for drinking water?

Own inside pipe	6	Stored water not connected to home	2
Shared inside pipe	5	Pipe outside of yard	1
Own pipe in yard	4	River or well/Other	0
Shared pipe in yard	3		

Crowding

22. No. of Children under 18 yrs. in the household _________________ (*refer to Ques#1*)

23. No. of people over ≥ 18 yrs. (share food and sleep in the home ≥ 4 d/week) __________(*refer to Ques#1*)

24. No. of rooms in household (total) __________________
(Do not count bathroom or kitchen unless can sit in kitchen and eat)
(Count veranda if totally enclosed and someone can sleep there)

Possessions

25. I'm going to read a list of items. Please say if there is one in your home in working condition.

	Yes	No
Oil, Gas or electric stove	1	0
Fridge	1	0
Washing machine	1	0
Living room set	1	0
Clothes Iron	1	0
Radio/Component Set	1	0
CD or DVD Player	1	0
TV	1	0
Cable (TV)/Android Box/Ready TV	1	0
Phone (Cell Phone/ Landline)	1	0
Computer (desktop or laptop)	1	0
Tablet	1	0
Bicycle (***not*** *child's*)	1	0
Motor bike	1	0
Motor car	1	0

Parenting Attitudes

Say*, "Now I am going to ask you whether you agree or disagree with some statements about looking after young children. There are no right or wrong answers, we just want to know what you think or feel. You can also ask me to repeat the statements".*

1. Too much love and attention will spoil a child.
   agree completely   agree a little bit   disagree a little bit   disagree completely

2. A parent needs to spank or beat young children when they are rude or they will grow up to be bad.
   agree completely   agree a little bit   disagree a little bit   disagree completely

3. It is important that a busy mother spend plenty time talking with her little baby.
   agree completely   agree a little bit   disagree a little bit   disagree completely

4. It is important that parents look at picture books with children less than 2 years old.
   agree completely   agree a little bit   disagree a little bit   disagree completely

5. The best way to get a child to behave is to praise him when he is good
   agree completely   agree a little bit   disagree a little bit   disagree completely

6. It is important that a busy mother should spend plenty time playing with her young child.
   agree completely   agree a little bit   disagree a little bit   disagree completely

7. There is no need to give toys to children less than 1 year old.
   agree completely   agree a little bit   disagree a little bit   disagree completely

8. Children should not be given crayons until they are ready to learn to write
   agree completely   agree a little bit   disagree a little bit   disagree completely

9. Young children should not be held when they cry because they this will make them want to be held all the time
   agree completely   agree a little bit   disagree a little bit   disagree completely

10. How a parent behaves with her child when s/he is young affects how well s/he will learn in school.
   agree completely   agree a little bit   disagree a little bit   disagree completely

CES- Short Depression Scale-Revised (Jamaica)

Say, *"This section is about how you might have been feeling or acting recently. I'm going to read some sentences which describe how persons sometimes feel. For each sentence, tell me if in* ***the past week*** *you felt or acted that way. Tell me if you felt that way* ***None of the time, A little of the time, some of the time or Most of the time.*** *Remember there are no right or wrong answers, only you can tell me how you feel".*

**In the past week** (from last __ till today)	None<br>*< 1 day*	A little<br>*1-2 d*	Some<br>*3-4 d*	Most<br>*5-7 d*
1. You were bothered by things that usually don't bother you.	0	1	2	3
2. You found it hard to think properly or concentrate.	0	1	2	3
3. You felt depressed.	0	1	2	3
4. You felt everything was hard to do.	0	1	2	3
5. You felt good about the future.	3	2	1	0
6. You felt afraid.	0	1	2	3
7. You did not sleep good.<br>*You tossed and turned, had difficulty falling asleep*	0	1	2	3
8. You felt happy.	3	2	1	0
9. You felt lonely.	0	1	2	3
10. You felt so tired you just sat around and did nothing	0	1	2	3

Parent Practices

Say, *"We're interested in knowing the kinds of things you and your children do in the home on a regular basis. I am going to ask some questions about activities you have been able to do with _____________."*

1. Have you ever made any toys or things for (*child's name*) to play with? Yes No
   *If **yes***, how many?_________
   Please describe what you made.
   ____
   ____

2. Are there other toys (bought, gift, siblings', made by others) in your home that (*child's name*) can play with? Yes No
   *If **yes***, how many toys?__________
   Please describe them for me.
   ____
   ____

3. In the past week, did you play with (*child's name*) with a toy or other object? Yes No
   *If **yes***, how many days? __________
   What did you do/play? ______________________________________
   ____

4. In the past week, did you play any games or other activities with (*child's name*) that did not need any toys (or tablet or phone) (e.g. peek-a-boo, clap hands, action songs, dance)? Yes No
   *If **yes***, how many days? __________
   What did you do? ____
   ____

5. Are there any picture books or children's books with pictures in your home for (*child's name*) to look at? Yes No
   *If **yes**,* how many? __________

6. In the past week, did you read or look at pictures in a book (or tablet) with (*child's name*)? Yes No
   *If **yes**,* how many days? __________

7. In the past week, did you tell (*child's name*) any stories (made up or based on other story)? Yes No
   *If **yes***, how many days? __________

What stories? ____

8. In the past week, did you tell (*child's name*) the names of people or things in the home or outside? Yes No

If ***yes***, how many days? __________

9. In the past week, did you sing with (*child's name*)? Yes No

If ***yes****,* how many days? __________

What songs did you sing? _________________________________

____

10. In the past week, did you give (*child's name*) a crayon(s)/pencil and help him/her to scribble or draw? Yes No

If ***yes****,* how many days? __________

11. Parents are often very busy. When you are working in the house, like cleaning or washing, do you chat with (*child's name*) about what you are doing?

None of time a little of the time sometimes most of the time

12. When (*child's name*) speaks or makes sounds do you respond or speak back to him/her?

None of time a little of the time sometimes most of the time

13. Do you and (*child's name*) have conversations or chat together – like make sounds or speak back and forth to each other?

None of time a little of the time sometimes most of the time

14. In the past week, did *(child's name)* do anything that pleased you or made you happy? Yes No

If ***yes***, what did you do when this happened?

(do not read responses, circle all that apply)

Nothing hugged/physical praised child give child something

15. When (*child's name*) behaves well or does something good how often do you praise him/her?

None of the time a little of the time sometimes often/most of the time

Child's Anthropometry

Weight __________________ (kg)

Length __________________ (cm)

Date of measurement:_____________________(day/month/year)

Interviewer ____________________ **Date** __________________ **(dd/mm/yyyy)**

Health Centre ID#________________ **Subject ID#** ______________

Locator information

Child's name ____________________________

Mother's name _____________________________ Pet name __________________

Mother's current address and directions ____

____

____

Current phone # ____________________

Any other phone # ___________________

Would it be ok for us to send you text messages? Yes _____ No _____

Other contact information (father of child, other caretaker)

Name __________________________________ Relationship ________________

Address _____________________________

Phone # _____________________________

Contact information of other close family or friend

Name __________________________________ Relationship ________________

Address _____________________________

Phone # _____________________________

Are you planning to move in the next year? Yes/No

If yes, do you know where you are going to move to? _________________________

Supervisor Checklist - CHA Home Visits and Phone Calls

Supervisor: ________________ Parish:_________________ CHA: ____________________

First Name of Child: ____________________________ Visit Month (#) ________________

Date of visit:___________________ Time of visit (Start: __________) (End: ____________)

Date of phone call: ____________________ Time of call (Start: __________) (End: ____________)

Adult worked with at home visit or on phone call *(relationship of the adult to child)*:

____

For Home Visits:

Last Visit (Review)

1. Visitor asked the caregiver to demonstrate with the child what had been done since the last visit	Caregiver demonstrated or explained all of the activities	Visitor assisted caregiver to remember some activities	Visitor did not ask
2. CHA asks caregiver about text message – if she read it and if she has any questions	□ Asked and discussed message	□ Asked if received only with no discussion	□ Did not ask about text messages

This Visit

3. Visitor prepared for the visit in advance and used appropriate materials	Very well	Adequately	A little	Not at all
4. Visitor *explained* the activities and objectives (what the child should achieve) to the *caregiver*	Very well	Adequately	A little	Did not explain
5. Visitor *demonstrated* the activities to the *caregiver and child*	Very well	Adequately	A little	Did not demonstrate
6. Visitor asked the caregiver *to do* the activities together with the child	Most of the time	Some of the time	A few times	Did not ask
7. Visitor *got feedback* from the caregiver on what she/he was going to do in the next week	Visitor and caregiver reviewed all activities	Visitor and caregiver reviewed some activities	Visitor and caregiver reviewed few activities	Did not review any of the activities

Relationship between Visitor and caregiver

8. Visitor listened and was responsive to the caregiver	Most of the time	Some of the time	Little of the time	No time at all
9. Visitor asked the caregiver about activities she/he does with the child which are similar to the current visit activities	Most of the time	Some of the time	Little of the time	No time at all
10. Visitor encouraged and positively reinforced the caregiver	Most of the time	Some of the time	Little of the time	No time at all
11. Overall, the relationship between the Visitor and Caregiver was warm, understanding and cooperative.	Most of the time	Some of the time	Little of the time	No time at all

Relationship between Visitor and child

12. Visitor gave child enough time to explore the materials	Most of the time	Some of the time	Little of the time	No time at all
13. Visitor listened to the child and responded to his/her vocalizations/gestures	Most of the time	Some of the time	Little of the time	No time at all
14. Visitor praised the child when he/she attempted/completed an activity	Most of the time	Some of the time	Little of the time	No time at all
15. Visitor talked about the activities that the child was doing	Most of the time	Some of the time	Little of the time	No time at all

Overall visit: Participation

16. The overall attitude of the Visitor during the visit was	Sharing	Some sharing	Insufficient participation	Dominating
17. The overall atmosphere of the visit was	Very happy	Happy	Neutral	Unhappy, uncomfortable

For Phone Calls

Introduction				
1	**CHA introduces herself**	◻ Yes	◻ No	
2	**CHA checks caregiver has time for call**	◻ Yes	◻ No	
**Review of activities**				
3	**CHA asks caregiver about activities done since last call**	◻ *Asks about both activities by name or description*	◻ *Asks about one activity by name or description*	◻ *Does not ask or no activity specified*
4	**CHA asks if baby can do the activities**	◻ *Asks and responds with encouragement and positive feedback*	◻ *Asks but does not respond*	◻ *Does not ask*
5	**CHA asks caregiver if any difficulties with activities. If caregiver did not do one or both activities CHA asks why**	◻ *Asks and responds with encouragement and possible solutions*	◻ *Asks but no follow-up feedback or solutions*	◻ *Does not ask*
**New activities**				
6	**CHA has <u>prepared for the call</u> and has selected and read appropriate activities**	◻ *Fully prepared,*<br><br>*has handbook, log book and script. Chose correct activities*	◻ *Somewhat prepared*	◻ *Not prepared*
7	**After reading <u>play activity CHA describes material</u> needed and allows time for caregiver to get**	◻ *Describes materials and suggests alternatives and checks that* caregiver *has/can get them*	◻ *Tells caregiver materials needed but does not provide support*	◻ *Does not talk about material*
8	**CHA asks caregiver if she <u>does anything similar</u> with child and responds with encouragement and praise**	◻ *Asks and responds with praise and encouragement*	◻ *Asks but no feedback*	◻ *Does not ask*
9	**CHA reminds caregiver to <u>let baby explore</u> and play with materials**	◻ *Reminds with encouragement*	◻ *Reminds with no encouragement*	◻ *Did not do*

10	**CHA <u>explains the play activities</u> well and asks caregiver if she has any questions or comments**	□ *Explains well with other suggestions*<br><br>- encourages caregiver to talk to baby about what they are doing<br><br>-To use concept words<br><br>*- Ask if any questions*	□ *Explains activities adequately but did not ask if caretaker had any questions*	□ *Only reads activity*	□ *Did not do*
11	**CHA reads and explains <u>language activity</u>, and asks caregiver how she would do the activity**	□ *Reads and explains activity well*<br><br>*- Ask caregiver for examples of what she could talk about, etc.*	□ *Reads and explains activity but gets little input from caregiver*	□ *Reads language activity only*	□ *Did not do*
12	**CHA encourages caregiver <u>to try both activities</u> while on the phone, with child and asks about how it goes**	□ *Encourages caregiver to try activity and ask how it goes*	□ *Ask caretaker to try activities but no follow up*	□*Did not ask caregiver to try activities*	□ *Child not available*
13	**CHA <u>did recap</u>. Gets feedback from caregiver on what she is going to do in the next 2 weeks**	□ *CHA and caregiver reviewed both activities*	□ *Reviewed one activity*	□ *Did not recap*	
**Text message and End**					
14	**CHA asks caregiver about <u>text message</u> – if she read it and if she has any questions**	□ *Asked and discussed message*	□ *Asked if received only with no discussion*	□ *Did not ask about text messages*	
15	**<u>Ends call</u> with encouragement and reminder that she will call again in 2 weeks**	□ *Very well*<br><br>- encouragement given or parenting tip with reminder about call	□ *Adequately*<br><br>*- Only reminds about call*	□ *Did not do*<br><br>*- No encouragement or reminder*	

**Overall Rating**					
**16**	**CHA <u>listens</u> carefully, does not interrupt caregiver, acknowledges what caregiver says and responds to questions/comments**	□ *Most of the time*	□ *Some of the time*	□ *Little of the time*	□ *None of time*
**17**	**CHA <u>responses</u> and comments are helpful, positive and encouraging**	□ *Most of the time*	□ *Some of the time*	□ *Little of the time*	□ *None of time*
**18**	**CHA reminds caregiver to <u>praise</u> baby for doing activity or trying**	□ *Most of the time*	□ *Some of the time*	□ *Little of the time*	□ *None of time*

Appendix 2

CONSENT FORM

Reach Up early childhood parenting programme: Evaluation of Implementation and Benefits in Jamaica, 2022

Dear Parent,

We are doing a study of a new programme in health centres and looking at how it affects children and mothers. In the programme a community health aide will visit your home and call you every two weeks to show you ways to help your child develop well. She will show you or describe play activities and let you practice doing them with your child. We may also send you text messages with tips and reminders. Some parents will start the programme now and others will start the programme after 8 months. All centres will continue to provide the usual health care for you and your child.

If you agree to take part in the study, we will administer a questionnaire to find out about your housing, education, how you have been feeling recently, and what you think about how children develop. This will take about 30 minutes. We will record your child's weight and length. We will repeat the questionnaire and measurements 8 months from now and also measure his/her development. **All information collected will be treated as confidential.** There are minimal risks to you and your child from participating. The only benefit to you is having an assessment of how your child is developing. If your child is developing more slowly than expected, with your permission, we will notify the clinic doctor so that your son/daughter can be referred for any assistance available.

You and your child's participation in this study are voluntary and you can choose to stop taking part at any time. If you choose not to participate this will in no way affect any access to health care. If you have any questions about the study please contact Professor Susan Walker at The Epidemiology Research Unit, Caribbean Institute for Health Research, U.W.I., Telephone 876 977- 6151-2.

For independent advice on your rights as a research participant please contact Professor Helen Trotman-Edwards, Chair-Mona Campus Research Ethics Committee, University of the West Indies, Mona, Kgn 7 Tel: (876) 970-4892, e-mail: mcrec@uwimona.edu.jm.

Reach Up early childhood parenting programme: Evaluation of Implementation and Benefits in Jamaica, 2022

The study has been explained to me and I have been given time to read this form. I understand what will be done. I have been given time to decide whether to participate. I will be given a copy of this form.

I AGREE ☐ DO NOT AGREE ☐ for my child and I to participate in this study.

Name of Participant: ___________________________________

Signature of Participant:________________________________

Date:___________________

Name of Researcher:___________________________________

Signature of Researcher: ________________________________

Date ___________________

Signature of Independent Witness _________________________________

Statistical analysis plan

Data Analysis Plan (on page 8 of the Trial protocol)

Impact evaluation

Primary analyses for the impact evaluation will be by intention to treat. We will compare the groups on baseline measurements and will control for any measures which differ among the groups at endline in multivariate analyses of outcome.

Following the primary regression analyses we will investigate whether impact varies by maternal characteristics (education, depressive symptoms) or child characteristics (age on enrolment). We will also investigate the mechanism for impact to determine if change in parent practices mediates the impact on child outcomes.
