# Supplementary material for: Blended Delivery of a Primary Care Parenting Program for Child Development: A Randomized Clinical Trial
Source: JAMA Netw Open. 2026 Feb 3;9(2):e2556024. doi: 10.1001/jamanetworkopen.2025.56024 (PMC12869336; doi:10.1001/jamanetworkopen.2025.56024)
Supplement: Supplement 2. — eTable 1. Estimated Annual Intervention Costs in USD Using 2022 World Bank Conversion Rate for Jamaican Dollars eTable 2. Benefit-Cost Ratio—Sensitivity Analysis eReferences [file jamanetwopen-e2556024-s002.pdf]

Supplementary Online Content

Chang SM, Smith JA, Wright AS, et al. Blended delivery of a primary care parenting program for child development: a randomized clinical trial. *JAMA Netw Open*. 2026;9(1):e2556024. doi:10.1001/jamanetworkopen.2025.56024

eTable 1. Estimated Annual Intervention Costs in USD Using 2022 World Bank Conversion Rate for Jamaican Dollars

eTable 2. Benefit-Cost Ratio—Sensitivity Analysis

eReferences

This supplementary material has been provided by the authors to give readers additional information about their work.

© 2026 Chang SM et al. *JAMA Network Open*.

eTable 1 – **Estimated Annual Intervention Costs in USD Using 2022 World Bank Conversion Rate for Jamaican Dollars**

**Description**	**Cost, $**	
	**Annual**	**Per child**
Variable costs	60 950.81	146.52
Supervisor wage, midwife[a]	1584.82	3.81
Supervisor wage, PHN[a]	2687.75	6.46
CHW wage[a]	22 046.66	53.00
Materials for families and CHWs[b]	29 133.40	70.03
Cost of telephone calls and text messaging to families	5498.17	13.22
Fixed costs	38 334.78	92.24
Supervisor training, PHNs and midwives[c]	24 524.10	58.95
CHW training[c]	13 810.68	33.29
Materials for health district[d]	2924.58	7.03
Program total	102 210.16	245.79

Abbreviations: CHW, community health worker; PHN, public health nurse.
[a]Nurses and CHWs are full-time employees of the Ministry of Health & Wellness. Costs are estimated for time spent on program activities, using salary scales provided by the Ministry of Health & Wellness: midwives and PHNs, 1 supervisory visit per month (1-hour visit, 30 minutes of travel time) and 1 hour per month for supervision in the clinic to supervise 4 CHWs (total, 2.5 hours per month); CHWs, 1 hour for a home visit with 30 minutes of travel time or 30 minutes for a telephone call (2.0 hours monthly per child; total, 8 hours per month).
[b]Printing cost for parent handbook for families, CHW visit record books for each family, and CHW travel bags, plus the cost of play materials provided over 1 year (printing of picture books and other materials, manufacturing of blocks).
[c]Training costs (venue, accommodations, meals, and transportation) for initial 10-day training and 3-day training.
[d]Printing manuals and curricula, supplies for toy making, storage cabinet, and laminating machines. The latter 2 items were estimated to last 5 years; thus, the actual cost was divided by 5.

© 2026 Chang SM et al. *JAMA Network Open*.

eTable 2 – **Benefit-Cost Ratio – Sensitivity Analysis**

This table presents the sensitivity analysis of the benefit-cost (BC) ratio under varying assumptions. We report BC ratios using three discount rates (3%, 5%, and 7%) and two wage benchmarks (minimum and average wages). For each combination, we provide lower and upper bounds based on the confidence intervals of the treatment effect, with 22% and 28% used as the respective bound of the confidence interval following Gertler et al. (2014). The analysis assumes constant returns as in Barnett and Masse (2007). As expected, higher discount rates reduce the BC ratio, while using average wages increases the ratio relative to minimum wages.

Benefit-Cost Ratios under different assumptions for Discount Rates and Wage Levels

Discount rate	Lower Bound		Mean		Upper Bound	
	Min. wage	Avg. wage	Min. wage	Avg. wage	Min. wage	Avg. wage
**3%**	14.1	20.3	16.0	23.1	18.0	25.9
**5%**	7.5	10.9	8.6	**12.3**	9.6	13.8
**7%**	4.3	6.2	4.9	7.0	5.4	7.9

***Sources**:* Average wage estimation as described in the text (page 12). Minimum wage data for 2022 are obtained from the World Bank (2024). Exchange rate information is based on World Bank (2022).

© 2026 Chang SM et al. *JAMA Network Open*.

eReferences

Barnett, W. Steven, and Leonard N. Masse. Comparative benefit–cost analysis of the Abecedarian program and its policy implications. Economics of education review 26, no. 1 (2007): 113-125.

Gertler P, Heckman J, Pinto R, et al. Labor market returns to an early childhood stimulation intervention in Jamaica. Science. 2014;344(6187):998-1001.

World Bank. *Official Exchange Rate (LCU per US$, period average) – Jamaica*. World Bank, 2022.

World Bank. *Jamaica Jobs Diagnostic*. Jobs Working Paper; Issue No. 83. Washington, D.C.: World Bank Group, 2024. Available at: http://documents.worldbank.org/curated/en/099710207022436976

© 2026 Chang SM et al. *JAMA Network Open*.
