# Supplementary material for: Blended Delivery of a Primary Care Parenting Program for Child Development: A Randomized Clinical Trial
Source: JAMA Netw Open. 2026 Feb 3;9(2):e2556024. doi: 10.1001/jamanetworkopen.2025.56024 (PMC12869336; doi:10.1001/jamanetworkopen.2025.56024)
Supplement: Supplement 3. — Data Sharing Statement [file jamanetwopen-e2556024-s003.pdf]

Data Sharing Statement

Chang. Blended Delivery of a Primary Care Parenting Program for Child Development. *JAMA Netw Open*. Published February 03, 2026. doi:10.1001/jamanetworkopen.2025.56024

Data
Additional Information: Measuring the benefits of the Reach Up early childhood parenting programme in Jamaica https://doi.org/10.1186/ISRCTN11059214 Registry - ISRCTN 11059214
Data available: No
